# Global High‐Risk *Escherichia coli* Clones in Moroccan Aquatic Settings: Genomic Evidence of Environmental Dissemination

**DOI:** 10.1155/ijm/6271074

**Published:** 2026-06-18

**Authors:** Amine Aiddi, Ilham Zerdani, Aboubakr Khazaz, Hafsa Mguild, Adil El Hamouchi, Kaotar Nayme

**Affiliations:** ^1^ Microbiology Unit, Laboratory of Ecology and Environment, Department of Biology, Faculty of Sciences Ben M′sik, Hassan II University, Casablanca, Morocco, uh2c.ac.ma; ^2^ Molecular Bacteriology Laboratory, Institut Pasteur du Maroc, Casablanca, Morocco, pasteur.ma; ^3^ Microbiology and Antimicrobial Agents Research Team, LB2VE, Department of Biology, Faculty of Sciences, Chouaib Doukkali University, El Jadida, Morocco, ucd.ac.ma; ^4^ Research Laboratory in Microbiology, Infectiology, Allergology and Pathogenic Surveillance (Larmias), Mohammed VI University of Sciences and Health (UM6SS), Casablanca, Morocco, um6ss.ma; ^5^ Genomic Sequencing Laboratory, Institut Pasteur du Maroc, Casablanca, Morocco, pasteur.ma

**Keywords:** aquatic environments, *Escherichia coli*, high-risk clones, One Health, whole-genome sequencing

## Abstract

**Background:**

Globally disseminated high‐risk *Escherichia coli* (*E. coli*) clones are major drivers of multidrug resistance (MDR) and severe clinical infections. Their presence in aquatic ecosystems represents a growing One Health concern, particularly in regions where wastewater management remains limited.

**Objective:**

This study aimed to investigate the environmental circulation and genomic characteristics of high‐risk *E. coli* in Moroccan aquatic environments.

**Methods:**

Water samples were collected between February and June 2024 from seven major rivers and three wastewater treatment plants (WWTPs) across eight Moroccan cities. A total of 22 *E. coli* isolates were recovered and analyzed by whole‐genome sequencing to determine their sequence types (STs), antimicrobial resistance genes, virulence factors, and plasmid replicon profiles.

**Results:**

Sixteen isolates originated from rivers (72.7%) and six from WWTPs (27.3%). Thirteen distinct STs were identified, dominated by the globally disseminated high‐risk clones ST44 (*n* = 4; 18.2%), ST410 (*n* = 4; 18.2%), and ST131 (*n* = 3; 13.6%). The most frequent extended‐spectrum *β*‐lactamase (ESBL) gene was *bla*_CTX-M-15_ (*n* = 13; 59.1%). Carbapenemase genes were found in five isolates (22.7%), including *bla_NDM-5_* (*n* = 2; 9.1%), *bla_OXA-48_* (*n* = 1; 4.5%), *bla_OXA-181_* (*n* = 1; 4.5%), *bla_OXA-484_* (*n* = 2; 9.1%), and *bla_OXA-244_* (*n* = 1; 4.5%), whereas *mcr-1.1* was detected in one isolate (4.5%). Plasmid profiling showed a predominance of IncF‐type replicons (IncFIB 81.8%, IncFII 77.3%, and IncFIA 68.2%), along with IncI (36.4%), IncX (13.6%), and IncHI (9.1%).

**Conclusion:**

This study provides the first genomic evidence of clinically relevant high‐risk *E. coli* clones and mobile resistance determinants circulating in Moroccan aquatic environments. These findings highlight the urgent need to strengthen integrated One Health surveillance to prevent environmental dissemination and reduce the public health impact of antimicrobial resistance.

## 1. Introduction

Antimicrobial resistance (AMR) has emerged as one of the most pressing global health challenges, undermining the effectiveness of antibiotics that are essential for treating bacterial infections [1]. In 2019, AMR was responsible for an estimated 1.27 million deaths worldwide, with the greatest impact observed in low‐ and middle‐income countries (LMICs) [2]. Among Gram‐negative bacteria, multidrug‐resistant (MDR) Enterobacterales have gained particular attention due to their ability to acquire and disseminate resistance determinants, including extended‐spectrum *β*‐lactamase (ESBL), ampicillinase C (AmpC), and carbapenemase genes [3]. This evolution has severely limited treatment options, forcing clinicians to rely on last‐resort antibiotics.

Within this group, *Escherichia coli* (*E.coli*) occupies a central position as both a commensal organism and a major opportunistic pathogen responsible for extraintestinal infections, notably urinary tract, and bloodstream infections [4]. Its ubiquity across human, animal, and environmental niches facilitates the acquisition and dissemination of resistance genes, making it a valuable sentinel species for AMR surveillance [5]. Over the past two decades, the global spread of high‐risk *E. coli* clones, including ST131, ST410, ST648, and ST38, has accelerated the transmission of resistance, primarily through plasmid‐mediated *β*‐lactamase genes such as *bla*_CTX-M_, *bla*_NDM_, and *bla*_OXA-48-like_ [6]. In Morocco, hospital‐based outbreaks involving *E. coli* strains producing NDM‐1, OXA‐48, and CTX‐M‐15 have been increasingly reported, in both clinical and community‐acquired infections [7], indicating that high‐risk lineages are already established in healthcare settings.

Although AMR surveillance has largely focused on clinical contexts, emerging evidence highlights the environment, particularly aquatic systems, as a critical reservoir for antibiotic resistance genes (ARGs) [8]. Aquatic ecosystems receiving wastewater discharges from urban, industrial, and hospital sources serve as hotspots for the accumulation and dissemination of antibiotic‐resistant bacteria (ARB) [9]. These ecosystems harbor complex microbial communities where resistant bacteria, residual antibiotics, and mobile genetic elements (MGEs), such as plasmids and integrons, create ideal conditions for horizontal gene transfer (HGT) [10]. Several international studies have confirmed the presence of MDR *E. coli* in environmental waters, underlining the risk of AMR transmission through environmental exposure routes [11] [12].

However, data from LMICs, including Morocco, remain limited [13]. Inadequate wastewater treatment infrastructure, especially in urban centers, frequently leads to the release of untreated or poorly treated effluents into surface waters used for domestic, agricultural, or recreational purposes [14]. Moreover, the scarcity of molecular studies examining resistance mechanisms in environmental *E. coli* isolates hinders our understanding of their evolution, adaptation, and dissemination within aquatic ecosystems.

To address this gap, the present study was conducted as part of a pilot investigation on environmental AMR in Morocco, focusing on the occurrence and genomic characteristics of *E. coli* isolated from wastewater and surface waters across several regions. Using whole‐genome sequencing (WGS), we characterized their resistome, virulome, plasmid content, and phylogenetic background to assess the potential migration of high‐risk *E. coli* clones from clinical settings into aquatic environments. These findings provide new insights into the role of Moroccan water ecosystems as secondary reservoirs contributing to the persistence and dissemination of clinically relevant *E. coli* lineages.

## 2. Material and Methods

### 2.1. Sample Sites and Collection

**Figure 1:**
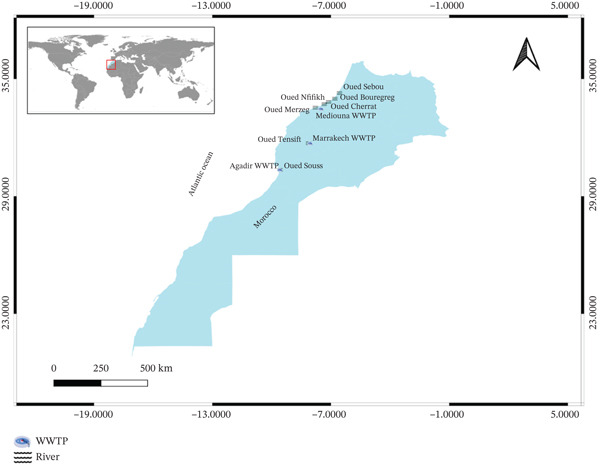
Map of Morocco showing the sampling sites.

A targeted water sampling campaign was conducted between February and June 2024 across eight Moroccan cities as part of a pilot study on environmental AMR surveillance. Sampling was performed monthly during the study period, with selected sites visited according to logistical accessibility and local environmental priorities. In total, 27 grab water samples were collected, 21 from seven rivers and six from three wastewater treatment plants (WWTPs) (Figure 1). This sampling approach aimed to assess spatial variability in bacterial contamination and resistance patterns across aquatic ecosystems exposed to varying levels of anthropogenic pressure.

#### 2.1.1. River Sampling

Seven major rivers were investigated: Oued sebou (Kenitra), Oued bouregreg (Rabat), Oued cherrat (Skhirat), Oued nfifikh (Mohammedia), Oued merzeg (Casablanca), Oued tensift (Marrakech), and Oued souss (Agadir), selected to represent diverse hydrological conditions and varying degrees of anthropogenic influence, including domestic, agricultural, and industrial discharges. To assess spatial differences in contamination, discrete grab samples were collected from three sites along each river (upstream, midstream, and downstream), reflecting gradients in pollutant exposure and potential bacterial dissemination. At each site, 500 mL of surface water was sampled at approximately 30‐cm depth using sterile glass containers.

#### 2.1.2. Wastewater Sampling

Three WWTPs located in Marrakech, Agadir, and Mediouna were included in this study. At each plant, grab samples were collected from both the untreated influent and the treated effluent to assess treatment efficiency and to characterize the potential persistence or release of resistant bacteria into receiving waters. This approach ensured the inclusion of key exposure points while enabling a comparative analysis of resistance profiles before and after treatment. Wastewater samples were collected using sterile 500‐mL polypropylene bottles.

To maintain integrity, all samples were stored at 4°C, transported to the laboratory in a covered icebox, and processed within 6 h of collection. Sampling procedures followed international standards, including ISO 19458:2006 and the Standard Methods for the Examination of Water and Wastewater [15, 16].

### 2.2. Bacterial Isolation and Identification

Water samples were processed for microbiological analysis using a selective enrichment approach. A 500‐*μ*L aliquot from each sample was inoculated into brain heart infusion (BHI) broth (Bio‐Rad) and incubated at 37°C for 24 h to promote bacterial growth. After incubation, 10 *μ*L of the enriched broth was streaked onto Brilliance UTI Clarity Agar (Oxoid), a chromogenic medium supplemented with ertapenem (0.5 *μ*g/mL) or ceftazidime (3 *μ*g/mL) for the selective isolation of carbapenem‐resistant and ESBL‐producing *E. coli*, respectively. Plates were incubated aerobically at 37°C for 18–24 h. The restreaking procedure was repeated until pure colonies were obtained. Colony morphology was evaluated according to the manufacturer′s recommendations, with *E. coli* typically producing dark pink to reddish colonies. Presumptive *E. coli* isolates were confirmed using the VITEK 2 system (bioMérieux, Marcy‐l′Étoile, France) and stored at −80°C for further analyses.

### 2.3. Antimicrobial Susceptibility Testing

The antimicrobial susceptibility profiles of all confirmed isolates were assessed using the standard disc diffusion method on Mueller–Hinton agar (Bio‐Rad), following the recommendations of the Antibiogram Committee of the French Society for Microbiology (CA‐SFM) (https://www.sfm-microbiologie.org/). The following antibiotics discs were used: ampicillin (10 *μ*g), amoxicillin–clavulanic acid (20/10 *μ*g), ticarcillin (75 *μ*g), cefoxitin (30 *μ*g), ceftazidime (10 *μ*g), cefotaxime (5 *μ*g), cefepime (30 *μ*g), aztreonam (30 *μ*g), ertapenem (10 *μ*g), imipenem (10 *μ*g), ciprofloxacin (5 *μ*g), ofloxacin (5 *μ*g), amikacin (30 *μ*g), gentamicin (10 *μ*g), tobramycin (10 *μ*g), and trimethoprim–sulfamethoxazole (1.25/23.75 *μ*g) (Oxoid Ltd., Hampshire, United Kingdom). Susceptibility to colistin was determined using the broth microdilution method, according to CA‐SFM recommendations (https://www.sfm-microbiologie.org/). The Double Disc Synergy Test (DDST) was performed to confirm ESBL production. Quality control was performed using the standard reference strain *E. coli* (ATCC 25922). Isolates exhibiting resistance to three or more antimicrobial classes were classified as MDR according to standardized criteria [17].

Isolates displaying reduced susceptibility to at least one carbapenem were tested using the NG‐Test CARBA 5 (NG Biotech, France). This immunochromatographic assay detects KPC, NDM, VIM, IMP, and OXA‐48‐like enzymes, following the manufacturer′s instructions [18].

### 2.4. DNA Extraction and WGS

Genomic DNA was extracted from overnight cultures of *E. coli* grown in Luria–Bertani (LB) broth (Bio‐Rad) at 37°C using the QIAamp DNA Mini Kit (Qiagen, Hilden, Germany) according to the manufacturer′s procedure. DNA concentration was measured using the Qubit 2.0 fluorometer (Thermo Fisher Scientific, USA), and purity was assessed with the ScanDrop^2^ spectrophotometer (Analytik Jena, Germany).

WGS was carried out on 22 *E. coli* strains at the Genomic Sequencing Laboratory, Institut Pasteur du Maroc, Casablanca, Morocco. Library preparation was performed using the Illumina DNA Prep Kit (Illumina, Eindhoven, Netherlands), and sequencing was conducted on the NextSeq 2000 platform (Illumina, San Diego, California, United States) using paired‐end reads (2 × 150 bp), targeting an average coverage depth of approximately 100×.

### 2.5. Bioinformatic Analysis

#### 2.5.1. Quality Control and Genome Assembly

The quality of generated paired‐end fastq reads was initially checked using FastQC (v0.12) (https://github.com/s-andrews/FastQC) and trimmed using trimmomatic (v0.39) (https://github.com/usadellab/Trimmomatic). High‐quality reads were then de novo assembled using SPAdes (v4.2.0) (https://github.com/ablab/spades). Assembly quality was evaluated with QUAST (v5.3.0) (https://github.com/ablab/quast), and contigs shorter than 500 bp were filtered out using BBMap (v39.01) (https://github.com/BioInfoTools/BBMap). The completeness and contamination of the final assemblies were assessed with CheckM (v1.2.2) (https://github.com/Ecogenomics/CheckM). Descriptive genome assembly statistics, including genome size, GC content, plasmid content, genome completeness, and contamination levels for all sequenced isolates, are provided in Table S1.

#### 2.5.2. Genomic Characterization and Phylogenetic Analysis

Species identification, MLST, serotype prediction, and detection of antimicrobial resistance genes were performed using Kleborate (v3.0.1) (https://github.com/klebgenomics/Kleborate). Virulence factors were screened using ABRicate (v1.0.1) (https://github.com/tseemann/abricate) against the Virulence Factor Database (VFDB). PlasmidFinder (v2.1) (https://cge.food.dtu.dk/services/PlasmidFinder/) was applied to identify plasmid replicon types, whereas MOB‐suite (v3.1.9) (https://github.com/phac-nml/mob-suite) was used to characterize plasmid structures and assess the association of resistance genes with plasmid sequences. Genome annotation was conducted through the Bacterial and Viral Bioinformatics Resource Center (BV‐BRC) (https://www.bv-brc.org).

A whole‐genome comparison of assemblies was performed using the alignment‐free mode of Split K‐mer Analysis (SKA2) (v0.5.0) (https://github.com/bacpop/ska.rust), which detects core‐genome single‐nucleotide polymorphisms (SNPs) based on k‐mer variation with accuracy comparable to alignment‐based approaches. The resulting SNP alignment was used to infer a maximum‐likelihood phylogeny in RAxML (v8.2.12) (https://github.com/stamatak/standard-RAxML) under the GTR + GAMMA substitution model with 1000 bootstrap replicates to estimate branch support.

#### 2.5.3. Data Visualization

The final phylogenetic tree was visualized using the iTOL platform v7 (https://itol.embl.de/). A comparative analysis of the genetic context of carbapenemase and *mcr1* encoding plasmids from EC90, EC114, EC121, and EC100 was carried out using BLAST Ring Image Generator (BRIG) v0.95 [19], aligning the reconstructed plasmid sequences with reference plasmids (GenBank accessions: MK291500.1, CP026476.1, CP045282.1, and KY471314.1).

## 3. Results

### 3.1. Bacterial Isolates and Antimicrobial Susceptibility Testing

A total of 22 *E. coli* isolates exhibiting resistance to extended‐spectrum cephalosporins and/or carbapenems were recovered from Moroccan aquatic environments, including river water (16/22, 72.7%) and WWTPs (6/22, 27.3%). Among the WWTPs isolates, four originated from untreated wastewater (4/6, 66.7%) and two from treated effluents (2/6, 33.3%) (Table 1). All isolates exhibited resistance to penicillins and third‐generation cephalosporins (22/22, 100%), including ampicillin, amoxicillin–clavulanic acid, ticarcillin, cefotaxime, and ceftazidime. Resistance to aztreonam and cefepime was also frequent, detected in 18/22 isolates (81.8%) and 19/22 isolates (86.4%), respectively, whereas cefoxitin resistance was observed in 9/22 isolates (40.9%).

**Table 1:** Antimicrobial resistance phenotypes and colistin MIC distribution among environmental *E. coli* isolates from Moroccan aquatic settings.

Strain	City	Sampling site	Phenotypic pattern of resistance	MIC of colistin (mg/L)
EC8	Agadir	Oued souss	AMP, AMC, TIC, CTX, CAZ, FEP, ATM, OFX, CIP	0.25
EC45	Mohammedia	Oued nfifikh	AMP, AMC, TIC, FOX, CTX, CAZ, FEP, ATM, ETP, OFX, CIP, TOB, GN, SXT	0.25
EC55	Casablanca	Oued merzeg	AMP, AMC, TIC, CTX, CAZ, FEP, ATM, TOB, AK, SXT	0.25
EC61	Mohammedia	Oued nfifikh	AMP, AMC, TIC, CTX, CAZ, FEP, ATM, OFX, CIP, TOB, AK, GN, SXT	0.25
EC80	Mediouna	WWTP mediouna (treated wastewater)	AMP, AMC, TIC, CTX, CAZ, FEP, ATM, OFX, CIP, TOB, GN, SXT	0.25
EC90	Rabat	Oued bouregreg	AMP, AMC, TIC, FOX, CTX, CAZ, FEP, ETP, IMP, OFX, CIP, SXT	0.25
EC91	Rabat	Oued bouregreg	AMP, AMC, TIC, FOX, CTX, CAZ, FEP, ATM, OFX, CIP, TOB, GN, AK, SXT	0.25
EC96	Skhirat	Oued cherrat	AMP, AMC, TIC, FOX, CTX, CAZ, FEP, ATM, OFX, CIP, TOB, GN, AK, SXT	0.25
EC97	Skhirat	Oued cherrat	AMP, AMC, TIC, FOX, CTX, CAZ, FEP, ATM, OFX, CIP, SXT	0.25
EC99	Agadir	WWTP agadir (untreated wastewater)	AMP, AMC, TIC, FOX, CTX, CAZ, OFX, CIP, SXT	0.25
EC100	Agadir	WWTP agadir (untreated wastewater)	AMP, AMC, TIC, CTX, CAZ, FEP, ATM, OFX, CIP, TOB, AK, GN, SXT, TOB, GN, SXT, COL	4
EC106	Agadir	WWTP agadir (treated wastewater)	AMP, AMC, TIC, CTX, CAZ, FEP, ATM, OFX, CIP, TOB, GN, SXT	0.25
EC113	Agadir	Oued souss	AMP, AMC, TIC, CTX, CAZ, FEP, ATM, OFX, CIP, TOB, GN, SXT	0.25
EC114	Agadir	WWTP agadir (untreated wastewater)	AMP, AMC, TIC, FOX, CTX, CAZ, FEP, ATM, ETP, IMP, TOB, GN, SXT	0.25
EC116	Agadir	Oued souss	AMP, AMC, TIC, CTX, CAZ, FEP, ATM, OFX, CIP, TOB, GN, SXT	0.25
EC119	Kenitra	Oued sebou	AMP, AMC, TIC, CTX, CAZ, FEP, ATM, OFX, CIP, TOB, GN, SXT	0.25
EC120	Kenitra	Oued sebou	AMP, AMC, TIC, CTX, CAZ, FEP, ATM, ETP, TOB, GN, SXT	0.25
EC121	Marrakech	WWTP marrakech (treated wastewater)	AMP, AMC, TIC, CTX, CAZ, FEP, ETP, TOB, GN, SXT	0.25
EC128	Marrakech	Oued tensift	AMP, AMC, TIC, FOX, CTX, CAZ, ATM, OFX, CIP, TOB, GN	0.25
EC135	Casablanca	Oued merzeg	AMP, AMC, TIC, FOX, CTX, CAZ, ATM, ETP, OFX, CIP, TOB, GN, SXT	0.25
EC141	Casablanca	Oued merzeg	AMP, AMC, TIC, FOX, CTX, CAZ, ATM, ETP, OFX, CIP, TOB, GN, SXT	0.25
EC146	Casablanca	Oued merzeg	AMP, AMC, TIC, CTX, CAZ, ATM, OFX, CIP, TOB, GN, SXT	0.25

Abbreviations: AK, amikacin; AMC, amoxicillin–clavulanic acid; AMP, ampicillin; ATM, aztreonam; CAZ, ceftazidime; CIP, ciprofloxacin; COL, colistin; CTX, cefotaxime; ETP, ertapenem; FEP, cefepime; FOX, cefoxitin; GN, gentamicin; IMP, imipenem; OFX, ofloxacin; SXT, trimethoprim–sulfamethoxazole; TIC, ticarcillin; TOB, tobramycin.

Resistance to fluoroquinolones was widespread, with 20 out of 22 isolates (90.9%) exhibiting reduced susceptibility to ofloxacin and ciprofloxacin. Aminoglycoside resistance was also frequent, notably against tobramycin and gentamicin (18/22, 81.8%). Nearly all isolates were resistant to trimethoprim–sulfamethoxazole (19/22, 86.4%), whereas reduced susceptibility to carbapenems (ertapenem or imipenem) was observed in 4/22 isolates (18.2%). Only one isolate EC100 exhibited a colistin MIC of 4 mg/L, indicating phenotypic resistance. The detailed antimicrobial susceptibility profiles are presented in Table 1.

### 3.2. MLST and Phylogenetic Analysis

**Figure 2:**
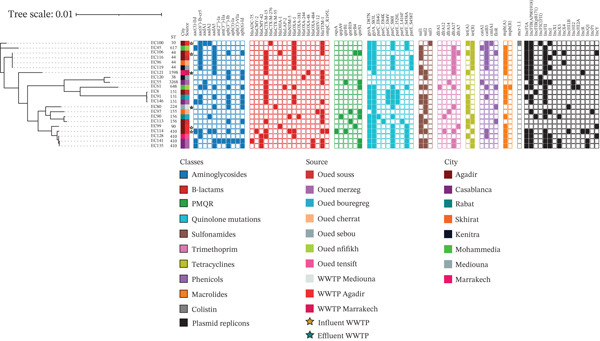
Phylogenetic tree of environmental *E. coli* isolates from Moroccan aquatic settings and their genomic features.

MLST analysis identified 13 distinct sequence types (STs) among the 22 *E. coli* isolates. The most frequent lineages were ST44 (*n* = 4) and ST410 (*n* = 4), followed by ST131 (*n* = 3) and ST156 (*n* = 2). The remaining isolates were assigned to single STs, including ST10, ST38, ST90, ST155, ST1598, ST224, ST617, ST648, and ST3268 (Figure 2).

Core genome SNP‐based phylogenetic reconstruction performed using SKA2 and RAxML revealed distinct clustering patterns consistent with MLST assignments. Cluster I (ST44) included four isolates (EC45, EC97, EC131, and EC141) and showed minimal intralineage variation, with the closest pair (EC116–EC119) differing by 56 SNPs. Cluster II (ST410) comprised four isolates (EC90, EC106, EC120, and EC135) and also exhibited low genomic divergence, with 158 SNPs between EC128 and EC135. Cluster III (ST131) contained three isolates (EC8, EC91, and EC113) with the smallest pairwise distance of 104 SNPs between EC8 and EC91. In contrast, isolates belonging to single‐occurrence STs were positioned on independent branches and displayed much larger SNP distances, reflecting higher genomic separation within the collection (Figure 2).

### 3.3. Resistome Profile

Whole‐genome analysis revealed a diverse resistome encompassing multiple antibiotic classes (Table 2). *β*‐lactam resistance genes were the most prevalent, with *bla_CTX-M-15_* (*n* = 13; 59.1%) as the dominant variant, followed by *bla_OXA-1_* (*n* = 11; 50.0%). Additional ESBL and AmpC genes were identified, including *bla_CMY-42_* (*n* = 3; 13.6%) and *bla_DHA-1_* (*n* = 2; 9.1%), as well as single detections of *bla_CMY-2_*, *bla_LAP-2_*, *bla_ADC-75_*, *bla_SHV-12_*, *bla_CTX-M-55_*, and *bla_CTX-M-276_* (each *n* = 1; 4.5%). Carbapenemase genes were also detected, notably *bla_NDM-5_* (*n* = 2; 9.1%), *bla_OXA-48_* (*n* = 1; 4.5%), *bla_OXA-181_* (*n* = 1; 4.5%), *bla_OXA-484_* (*n* = 2; 9.1%), and *bla_OXA-244_* (*n* = 1; 4.5%).

**Table 2:** Antibiotic resistance genes and plasmids replicons found in environmental *E. coli* isolates.

Strain	ST	Aminoglycosides	*β*‐lactams	Fluoroquinolones	Sulfonamides	Trimethoprim	Tetracyclines	Phenicols	Macrolides	Colistin	Plasmids
EC100	10	*aadA1; aadA2; aac(6* ^′^ *)-Ibcr5*	*blaCTX-M276; blaOXA-1; blaTEM-1*	*parC_S80I; parE_S458A; gyrA_D87N; gyrA_S83L; aac(6* ^′^ *)-Ib-cr5*	*sul3*	*—*	*—*	*cmlA1; catB3*	*—*	*mcr-1.1*	IncFIA; IncFIB(AP001918); IncFIC(FII); IncI
EC45	617	*aadA5; aac(3)-IId; aac(6* ^′^ *)-Ib-cr5*	*blaTEM-1; blaOXA-1*	*gyrA_D87N; gyrA_S83L; parE_S458A; parC_S80I; aac(6* ^′^ *)-Ib-cr5*	*sul1*	*dfrA17*	*tet(B)*	*catA1; catB3*	*mph(A)*	—	IncFIA; IncFIB(AP001918); IncFII
EC106	44	*aph(3* ^″^ *)-Ib; aph(6)-Id; aac(6* ^′^ *)-Ib-cr5; aadA5*	*blaDHA-1; blaCTX-M-15; blaOXA-1; blaTEM-1*	*qnrB4; qnrS1; gyrA_D87N; gyrA_S83L; aac(6* ^′^ *)-Ib-cr5*	*sul1*	*dfrA17; dfrA7*	*tet(A); tet(B)*	*floR; catB3*	*mph(A)*	—	IncFIA; IncFIB(AP001918); IncFII; IncX1; IncX4; IncY
EC116	44	*aadA5; aac(6* ^′^ *)-Ib-cr5*	*blaCTX-M-15; blaOXA-1*	*gyrA_D87N; gyrA_S83L; parE_S458T; parC_S80I; aac(6* ^′^ *)-Ib-cr5*	*sul1*	*—*	*tet(B)*	*catB3*	*mph(A)*	—	IncFIA; IncFIB(AP001918; IncFII; IncI
EC96	44	*aadA5; aac(6* ^′^ *)-Ib-cr5*	*blaCTX-M-15; blaOXA-1*	*gyrA_D87N; gyrA_S83L; parE_S458T; parC_S80I; aac(6* ^′^ *)-Ib-cr5*	*—*	*dfrA17*	*tet(B)*	*catB3*	*mph(A)*	—	IncFIA; IncFIB(AP001918); IncFII; IncX4
EC119	44	*aadA5; aac(6* ^′^ *)-Ib-cr5*	*blaCTX-M-15; blaOXA-1*	*gyrA_D87N; gyrA_S83L; parE_S458T; parC_S80I; aac(6* ^′^ *)-Ib-cr5*	*sul1*	*dfrA17*	*tet(B)*	*catB3*	*mph(A)*	—	IncFIA; IncFIB(AP001918); IncFII; IncI
EC121	1598	*aadA5; aph(6)-Id; aph(3* ^″^ *)-Ib*	*blaCTX-M-15; blaTEM-1; blaOXA-48*	*gyrA_S83L*	*sul1; sul2*	*dfrA17*	*tet(A)*	*—*	*mph(A)*	—	IncFIA; IncFIB(AP001918); IncFII; IncR
EC120	38	*aph(3* ^″^ *)-Ib; aph(6)-Id*	*blaTEM-1; blaOXA-244*	*—*	*sul2*	*—*	*—*	*—*	*—*	—	IncFIA; IncFIB(AP001918); IncFIC(FII)
EC55	3268	*aadA1*	*blaCTX-M-15; blaTEM-1*	*qnrS1; qnrB1*	*—*	*dfrA14*	*—*	*catA1*	*—*	—	IncFIB(AP001918); IncFIC(FII); IncFII; IncI; IncHI2
EC61	648	*aph(6)Id; aph(3* ^″^ *)Ib; aac(6* ^′^ *)-Ib-cr5; aadA5; aadA1*	*blaLAP-2; blaCTX-M-15; blaOXA-1*	*gyrA_D87N; gyrA_S83L; parE_S458A; parC_S80I; aac(6* ^′^ *)-Ib-cr5; qnrS*	*sul1; sul2*	*dfrA17; dfrA1*	*tet(A)*	*floR; catB3*	*mph(B); mph(A)*	—	IncFIA; IncFIB(AP001918); IncFII; IncHI2; IncHI2A
EC8	131	*—*	*blaCTX-M-15*	*parC_E84V; parC_S80I; parE_I529L; gyrA_D87N; gyrA_S83L*	*—*	*—*	*—*	*—*	*mph(A)*	—	IncFIB(pB171); IncFII
EC91	131	*aadA5; aph(6)-Id; aph(3* ^″^ *)Ib; aac(6* ^′^ *)-Ib-cr5*	*blaCTX-M-15; blaOXA-1;*	*gyrA_D87N; gyrA_S83L; parC_E84V; parC_S80I; parE_I529L; aac(6* ^′^ *)-Ib-cr5*	*sul1; sul2*	*—*	*tet(A)*	*catB3*	*mph(A)*	—	IncFIA; IncFIB(AP001918); IncFII
EC146	131	*aph(6)Id; aph(3* ^″^ *)Ib; aac(6* ^′^ *)-Ib-cr5; aac(3)-IId*	*blaCTX-M-15; blaOXA-1; blaTEM-1*	*gyrA_D87N; gyrA_S83L; parE_I529L; parC_E84V; parC_S80I; aac(6* ^′^ *)-Ib-cr5*	*sul1; sul2*	*—*	*tet(A)*	*—*	*mph(A)*	—	IncFIB(AP001918); IncFII
EC80	224	*aadA5; aph(6)-Id; aph(3* ^″^ *)-Ib*	*blaCTX-M-55*	*gyrA_D87N; gyrA_S83L; parC_S80I*	*sul2*	*dfrA17*	*tet(A)*	*floR*	*—*	—	IncH1B
EC97	155	*—*	*blaTEM-1; blaSHV-12*	*gyrA_S83L; qnrS1; qnrB19*	*sul2*	*dfrA14*	*tet(A)*	*—*	*—*	—	IncFIB(AP00198; IncFII; IncI; IncP1
EC90	156	*aadA2*	*blaTEM-1; blaOXA-181; blaNDM-5*	*parC_E84G; parC_S80I; gyrA_D87N; gyrA_S83L; qnrS1; qepA9*	*sul1*	*dfrA12*	*tet(B)*	*—*	*mph(A)*	—	IncFIB(pB171); IncFII; IncX3
EC113	156	*aph(6)-Id; aph(3* ^″^ *)-Ib*	*blaCTX-M-15; blaTEM-1*	*gyrA_D87N; gyrA_S83L; parE_L416F; parC_E84K; qnrB19*	*sul2; sul1*	*—*	*tet(A); tet(B)*	*floR*	*—*	—	—
EC99	90	*—*	*blaDHA-1*	*gyrA_D87N; gyrA_S83L; parC_S80I; parE_S458A; qnrB4*	*sul1*	*dfrA7*	*tet(A)*	*—*	*mph(A)*	—	IncY
EC114	410	*aac(6* ^′^ *)-Ib-cr5; aadA2; aadA5; aph(6)Id; aph(3* ^″^ *)Ib; aac(3)-IId*	*blaOXA-1; blaCMY-2; blaCTX-M-15; blaTEM1; ompC_R195L; blaNDM-5*	*aac(6* ^′^ *)-Ib-cr5; gyrA_D87N; gyrA_S83L; parC_S80I; parE_S458A; oqxA; qnrB*	*sul1; sul2*	*dfrA12; dfrA17*	*tet(B)*	*catB3*	*mph(A)*	—	IncFIA; IncFIB(AP001918); IncFII; IncH1B; IncR; IncQ1
EC128	410	*aac(6* ^′^ *)-Ib-cr5*	*blaCMY-42; blaCTX-M-15; blaOXA-1*	*parC_S80I; parE_S458A; gyrA_D87N; gyrA_S83L; aac(6* ^′^ *)-Ib-cr5*	*—*	*—*	*tet(A)*	*catB3*	*mph(A)*	—	IncFIA; IncFIB(AP001918); IncFII; IncI; IncY
EC141	410	*ant(3* ^″^ *)IIa; ant(2* ^″^ *)Ia; aac(3)-IId; aph(3* ^″^ *)Ib; aph(6)-Id; aadA5; aph(3* ^′^ *)Ia; aph(3* ^′^ *)-VIa*	*blaCMY-42; blaADC-75; blaOXA-484*	*parE_S458A; parC_S80I; gyrA_D87N; gyrA_S83L; qnrS1*	*sul2; sul1*	*dfrA17*	*tet(B)*	*—*	*mph(A)*	—	IncFIA; IncFIB(AP001918); IncFII; IncI; IncX1; IncP1
EC135	410	*aac(3)IId; aph(3* ^″^ *)Ib; aph(6)-Id; aadA5*	*blaCMY-42; blaOXA-484*	*parE_S458A; parC_S80I; gyrA_D87N; gyrA_S83L; qnrS1*	*sul2; sul1*	*dfrA17*	*tet(B)*	*—*	*mph(A)*	—	IncFIA; IncFIB(AP001918); IncFII; IncI; IncX1; IncP1

Aminoglycoside‐modifying enzyme genes were widespread, particularly *aadA5* (*n* = 12; 54.5%), *aac(6-Ib-cr5* (*n* = 11; 50.0%), *aph(3*  ^″^ *)-Ib* (*n* = 11; 50.0%), and *aph(6)-Id* (*n* = 11; 50.0%), with additional variants including *aac(3)-IId* (*n* = 5; 22.7%), *aadA1* (*n* = 3; 13.6%), *aadA2* (*n* = 3; 13.6%), *ant(2*  ^″^ *)-Ia* (*n* = 1; 4.5%), *ant(3*  ^″^ *)-IIa* (*n* = 1; 4.5%), *aph(3*  ^′^ *)-Ia* (*n* = 1; 4.5%), and *aph(3*  ^′^ *)-VIa* (*n* = 1; 4.5%).

Fluoroquinolone resistance was mediated by both plasmid‐borne and chromosomal determinants. Plasmid‐mediated *qnr* genes were observed in 13 isolates (59.1%), including *qnrS1* (*n* = 7; 31.8%), *qnrB1* (*n* = 2; 9.1%), *qnrB19* (*n* = 2; 9.1%), and *qnrB4* (*n* = 2; 9.1%). Mutations in the quinolone resistance–determining regions (QRDR) were highly prevalent, specifically *gyrA_S83L* (*n* = 19; 86.4%) and *parC_S80I* (*n* = 16; 72.7%).

Additional resistance determinants included *sul1* (*n* = 14; 63.6%) and *sul2* (*n* = 11; 50.0%) for sulfonamides; *dfrA17* (*n* = 11; 50.0%) for trimethoprim; *catB3* (*n* = 10; 45.5%), *floR* (*n* = 4; 18.2%), and *catA1* (*n* = 2; 9.1%) for phenicols; *mph(A)* (*n* = 16; 72.7%) for macrolides; and *tet(A)* and *tet(B)* (each *n* = 11; 50.0%) for tetracyclines. Colistin resistance was detected in one isolate EC100 carrying *mcr-1.1* (*n* = 1; 4.5%).

### 3.4. Plasmids Replicons

Plasmid replicon typing revealed a diverse plasmid repertoire among the 22 *E. coli* isolates (Table 2). IncF‐type plasmids were the most prevalent, including IncFIB (AP001918) (*n* = 18; 81.8%), IncFII (*n* = 17; 77.3%), IncFIA (*n* = 15; 68.2%), and IncFIC (FII) (*n* = 3; 13.6%). These replicons frequently co‐occurred within the same isolates, indicating multireplicon IncF backbones. Secondary plasmid groups included IncI (*n* = 8; 36.4%), IncX‐type plasmids: IncX1 (*n* = 2; 9.1%), IncX3 (*n* = 3; 13.6%), and IncX4 (*n* = 2; 9.1%), as well as IncHI1B (*n* = 2; 9.1%). Additional incompatibility groups were found at lower frequencies, including IncQ1 (*n* = 4; 18.2%), IncY (*n* = 4; 18.2%), IncHI2 (*n* = 2; 9.1%), IncR (*n* = 2; 9.1%), IncP1 (*n* = 1; 4.5%), and IncHI2A (*n* = 1; 4.5%).

Carbapenemase‐encoding genes were detected on distinct plasmid replicons among the three carbapenem‐resistant *E. coli* isolates (Table 2). The *bla_OXA-48_* gene in EC121 was located on an IncFII‐type plasmid, whereas *bla_NDM-5_* detected in EC90 and EC114 was associated with IncFIB‐type plasmids. In addition, EC90 carried an IncX3 plasmid coharboring *bla_OXA-181_* and *qnrS1*, illustrating the diversity of plasmid backbones involved in the mobilization of carbapenemase genes.

The colistin resistance gene *mcr-1.1* was identified on an IncI2 plasmid (*n* = 1; 4.5%), detected in EC100. Overall, the plasmid content of these isolates highlighted a wide distribution of incompatibility groups, encompassing conjugative and mobilizable elements associated with key resistance determinants.

### 3.5. Virulence Factors and Serotyping

**Figure 3:**
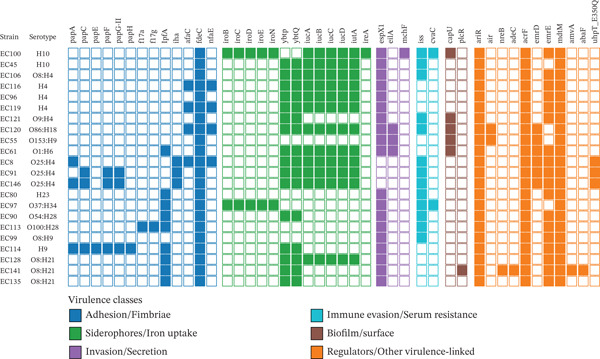
Distribution of virulence genes and serotypes among environmental *E. coli* isolates from Moroccan aquatic settings.

A total of 56 virulence associated genes were identified in the analyzed isolates (Figure 3). The detected genes were grouped into six functional categories, comprising adhesion and fimbriae, iron acquisition and siderophores, invasion and secretion, immune evasion, biofilm or surface structures, and regulatory or efflux‐associated determinants.

Adhesion and fimbrial genes were widely distributed, including *papA*, *papC*, *papF*, *papG-II*, *lpfA*, *fdeC*, *afaC*, *f17A*, *f17G*, *nfaE*, and *iha*. These were detected in nearly all isolates, with extended fimbrial clusters observed in EC91, EC114, and EC146. Iron uptake and siderophore‐related genes (*ybtP*, *ybtQ*, *iroB–E*, *iroN*, *iucA–D*, and *iutA*) were also common, particularly abundant in EC61, EC90, EC96, EC100, and EC106. Invasion and secretion genes (*eilA* and *espX1*) were consistently identified across the collection. Genes associated with immune evasion and serum resistance (*iss* and *cvaC*) were identified in over two‐thirds of the isolates, whereas biofilm and surface‐associated elements (*capU*) appeared sporadically. Regulatory and efflux‐linked virulence genes (*adeC*, *acrF*, *emrD*, *emrE*, *mdtM*, *amvA*, *abaF*, *air*, *ariR*, and *uhpT_E350Q*) occurred in nearly all isolates, often co‐occurring with other functional categories.

Serotyping identified 15 distinct O:H combinations among the isolates (Figure 3). The most recurrent serotypes were O25:H4 (*n* = 3), O8:H21 (*n* = 3), and H4 (*n* = 3), whereas others such as O8:H4, O9:H4, O86:H18, O153:H9, O1:H6, H10, H23, O37:H34, O54:H28, O100:H28, O8:H9, and H9 were detected in single isolates.

## 4. Discussion

In this study, the genomic diversity observed among the 22 *E. coli* isolates recovered from Moroccan aquatic environments highlights the coexistence of multiple high‐risk lineages within rivers and wastewater systems. MLST analysis revealed 13 distinct STs, with ST44, ST410, and ST131 being the most frequent, followed by ST156 and several sporadic types including ST10, ST38, ST617, ST648, and ST1598. The predominance of globally disseminated lineages such as ST410 and ST131 suggests that environmental waters act as reservoirs reflecting the clones circulating in clinical settings. ST410 is an emerging high‐risk clone frequently carrying carbapenemases and ESBL genes isolated not only from humans but also from wastewater and surface water sources [20]. Similarly, ST131 represents one of the most successful extraintestinal pathogenic *E. coli* (ExPEC) lineages, responsible for a significant proportion of urinary tract and bloodstream infections worldwide [21]. In Morocco, *E. coli* ST131 producing *bla_CTX-M-15_* was reported as the dominant clone among community and hospital‐associated urinary isolates [22], sharing identical resistance determinants with the environmental ST131 detected in this study. The presence of this lineage in both clinical and environmental contexts provides strong evidence of its continuous dissemination and persistence across human and aquatic compartments. Phylogenetic reconstruction confirmed the clustering of isolates within their respective STs, with limited SNP variation within major clades, supporting the hypothesis of recent common ancestry and local persistence of these clones across geographically distinct Moroccan aquatic sites. These findings are consistent with previous environmental studies showing close genomic relatedness between aquatic and clinical isolates [23], highlighting the role of aquatic settings as a critical interface in the circulation of MDR *E. coli* between human and environmental compartments.

Among all detected resistance determinants, ESBL genes were predominant, notably *bla*_CTX-M-15_ (*n* = 13) and *bla*_TEM-1_ (*n* = 13), followed by *bla*_OXA-1_ (*n* = 11). The dominance of CTX‐M‐15 aligns with its widespread occurrence among Moroccan clinical *E. coli* isolates in both hospital and community settings [24]. Its detection in both river and WWTP samples suggests that human‐derived discharges are contributing to the aquatic dissemination of ESBL‐producing clones. In addition, carbapenemase genes, including *bla*_NDM-5_, *bla*_OXA-48_, *bla*_OXA-181_, *bla*_OXA-244_, and *bla*_OXA-484_, were found in five isolates, reflecting the dominance of OXA‐48‐like and NDM variants previously reported in Moroccan clinical Enterobacterales isolates [7]. Their detection in aquatic samples highlights the persistence of these clinically derived carbapenemase genes in the environment and their potential role in the broader dissemination of carbapenem resistance. Beyond *β*‐lactams resistance, the plasmid‐mediated *mcr-1.1* gene detected in one isolate EC100 from a WWTP influent represents an alarming signal of emerging transferable colistin resistance within Moroccan aquatic environments. This finding extends the resistance spectrum observed in the present study and suggests that selective pressures in wastewater systems may favor the persistence of mobile elements conferring resistance to last‐resort antibiotics. Alongside this, several additional resistance genes such as *aadA5*, *aac(6*  ^′^ *)-Ib-cr5*, *qnrS1*, *sul1*, *tet(A/B)*, and *catB3*, together with chromosomal mutations in *gyrA* and *parC*, were detected in isolates from both rivers and WWTPs, reflecting the accumulation of multiple resistance mechanisms within these environments.

**Figure 4:**
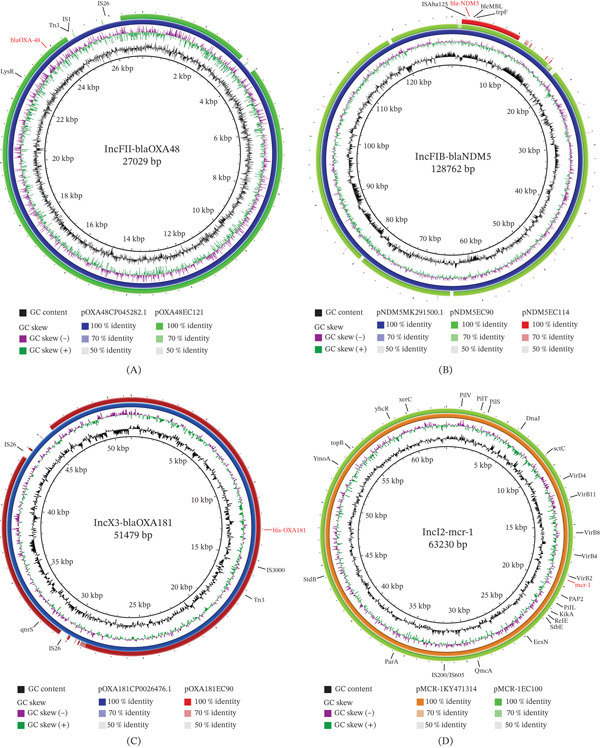
BLAST ring comparison of plasmids carrying resistance genes from environmental *E. coli* isolates recovered in this study and their corresponding reference sequences. Each ring represents a reconstructed plasmid sequence aligned to the reference plasmid at the center. (A) EC121—IncFII plasmid aligned with pOXA48 (accession: CP045282.1, Lebanon). (B) EC90 and EC114—IncFIB plasmid aligned with pNDM5 (accession: MK291500.1, Pakistan). (C) EC90—IncX3 plasmid aligned with pOXA181 (accession: CP026476.1, South Korea). (D) EC100—IncI2 plasmid aligned with pMCR1 (accession: KY471314.1, Argentina).

Plasmid replicon typing revealed diverse incompatibility groups among the *E. coli* isolates, dominated by IncF‐type backbones, IncFII, IncFIA, and IncFIB, which frequently coexisted within the same strains, indicating mosaic multireplicon structures typical of ESBL and carbapenemase producing *E. coli* (Figure 4). Carbapenemase genes were carried on distinct plasmids: *bla_OXA-48_* in EC121 was located on an IncFII plasmid identical to pCP045282.1 first reported from a clinical *E. coli* isolate in Lebanon [25]. In EC90 and EC114, *bla_NDM-5_* was associated with IncFIB plasmids highly similar to pMK291500.1 identified in *E. coli* from chicken meat in Pakistan [26]. EC90 also harbored *bla_OXA-181_* and *qnrS1* on an IncX3 plasmid closely related to pCP026476.1 originally described in a *E. coli* isolate from South Korea [27]. Furthermore, EC100, recovered from WWTP, carried *mcr-1.1* on an IncI2 plasmid sharing > 99% identity with pKY471314, previously detected in a bloodstream *E. coli* isolate from Argentina [28]. Taken together, these comparisons reveal a striking genomic convergence between plasmids circulating in Moroccan aquatic environments and plasmids previously reported from geographically distant regions and diverse ecological sources. The near‐identical backbone structures observed across these plasmids strongly suggest that globally disseminated resistance plasmids are circulating across human, animal, and environmental compartments. Such findings support the concept that aquatic ecosystems function as environmental interfaces where internationally distributed antimicrobial resistance plasmids can persist, accumulate, and potentially facilitate the exchange of clinically relevant resistance determinants within a One Health context.

The characteristics of virulence genes showed that a large proportion of the environmental *E. coli* isolates carried adhesion‐ and fimbriae‐associated genes (*papA*, *papC*, *papG-II*, *lpfA*, *fdeC*, and *afaC*), which are crucial for colonization and infection of host tissues [29]. Iron acquisition genes (*ybtP/Q*; *iroB*, *C*, *D*, *E*, and *N*; *iucA*, *B*, *C*, and *D*; and *iutA*) were also highly prevalent, particularly among isolates recovered from wastewater, emphasizing their importance in bacterial persistence under nutrient‐limited conditions [30]. Immune evasion genes (*iss* and *cvaC*) and efflux‐related determinants (*acrF*, *emrD*, and *mdtM*) were frequently detected, supporting the adaptation of these isolates to environmental stress. Similar virulence repertoires have been described in Moroccan clinical *E. coli* isolates responsible for urinary tract infections [22], suggesting a shared genetic background between environmental and clinical populations. The detection of serotypes O25:H4 and H4, commonly linked to the high‐risk clone ST131 [31], indicates that river and wastewater‐derived *E. coli* may contribute to the environmental circulation of virulent lineages with potential clinical relevance.

Several factors should be considered when interpreting these findings. This study was designed as a pilot genomic investigation and therefore included a limited number of environmental isolates, which limits broad population‐level conclusions regarding the distribution of clones, plasmids, and resistance determinants across Moroccan aquatic ecosystems. In addition, the limited geographic coverage may not fully capture the diversity of antimicrobial‐resistant *E. coli* circulating in aquatic environments across Morocco. Finally, genomic analyses were performed using short‐read sequencing technologies (Illumina), which enable reliable detection of resistance genes and plasmid replicons but may not fully resolve complex plasmid structures or the precise genetic contexts of MGEs. Future studies integrating long‐read sequencing technologies, such as Oxford Nanopore or PacBio, would facilitate more accurate plasmid reconstruction and localization of key resistance determinants.

## 5. Conclusion

This study provides the first genomic investigation of *E. coli* lineages circulating in Moroccan aquatic environments. The detection of globally distributed high‐risk clones, along with key resistance genes including ESBL, carbapenemases, and other resistance genes, underscores the convergence of clinically significant determinants within environmental reservoirs. The recovery of these isolates from both river and WWTPs reflects the continuous influence of anthropogenic activities in maintaining and spreading AMR beyond healthcare settings, underscoring the urgent need to strengthen integrated One Health surveillance and improve wastewater management practices to curb the environmental dissemination of MDR bacteria.

## Author Contributions

**Amine Aiddi:** conceptualization, writing – original draft, data curation, investigation, methodology, visualization, formal analysis. **Ilham Zerdani:** supervision, validation, writing – review and editing, project administration, methodology, resources. **Aboubakr Khazaz:** methodology. **Hafsa Mguild:** methodology. **Adil El Hamouchi:** methodology. **Kaotar Nayme:** supervision, validation, writing – review and editing, project administration, methodology, resources.

## Funding

This work was supported by the Centre National pour la Recherche Scientifique et Technique, 10.13039/501100006319, 8 UH2C2023.

## Conflicts of Interest

The authors declare no conflicts of interest.

## Supporting information


**Supporting Information** Additional supporting information can be found online in the Supporting Information section. Table S1: Genome assembly and quality metrics of the 22 sequenced *Escherichia coli* isolates, including genome size, GC content, plasmid content, genome completeness, and estimated contamination levels.

## Data Availability

The genome sequences generated in this study have been deposited in the DDBJ/ENA/GenBank databases under BioProject Accession Number PRJNA1395011.
